# Dietary fatty acids modulate oxidative stress response to air pollution but not to infection

**DOI:** 10.3389/fphys.2024.1391806

**Published:** 2024-05-09

**Authors:** Ann-Kathrin Ziegler, Johan Kjellberg Jensen, Lucía Jiménez-Gallardo, Jenny Rissler, Anders Gudmundsson, Jan-Åke Nilsson, Caroline Isaksson

**Affiliations:** ^1^ Department of Biology, Lund University, Lund, Sweden; ^2^ Centre for Environmental and Climate Science (CEC), Lund University, Lund, Sweden; ^3^ Department of Biodiversity, Ecology and Evolution, Complutense University of Madrid, Madrid, Spain; ^4^ Ergonomics and Aerosol Technology, Department of Design Sciences, Lund University, Lund, Sweden; ^5^ Bioeconomy and Health, RISE Research Institutes of Sweden, Lund, Sweden

**Keywords:** antioxidants, avian, immune function, tropospheric ozone, nutrition, multiple stressors

## Abstract

Anthropogenic changes to the environment expose wildlife to many pollutants. Among these, tropospheric ozone is of global concern and a highly potent pro-oxidant. In addition, human activities include several other implications for wildlife, e.g., changed food availability and changed distribution of pathogens in cities. These co-occurring habitat changes may interact, thereby modulating the physiological responses and costs related to anthropogenic change. For instance, many food items associated with humans (e.g., food waste and feeders for wild birds) contain relatively more ω6-than ω3-polyunsaturated fatty acids (PUFAs). Metabolites derived from ω6-PUFAs can enhance inflammation and oxidative stress towards a stimulus, whereas the opposite response is linked to ω3-derived metabolites. Hence, we hypothesized that differential intake of ω6-and ω3-PUFAs modulates the oxidative stress state of birds and thereby affects the responses towards pro-oxidants. To test this, we manipulated dietary ω6:ω3 ratios and ozone levels in a full-factorial experiment using captive zebra finches (*Taeniopygia guttata*). Additionally, we simulated an infection, thereby also triggering the immune system’s adaptive pro-oxidant release (i.e., oxidative burst), by injecting lipopolysaccharide. Under normal air conditions, the ω3-diet birds had a lower antioxidant ratio (GSH/GSSG ratio) compared to the ω6-diet birds. When exposed to ozone, however, the diet effect disappeared. Instead, ozone exposure overall reduced the total concentration of the key antioxidant glutathione (tGSH). Moreover, the birds on the ω6-rich diet had an overall higher antioxidant capacity (OXY) compared to birds fed a ω3-rich diet. Interestingly, only the immune challenge increased oxidative damage, suggesting the oxidative burst of the immune system overrides the other pro-oxidative processes, including diet. Taken together, our results show that ozone, dietary PUFAs, and infection all affect the redox-system, but in different ways, suggesting that the underlying responses are decoupled despite that they all increase pro-oxidant exposure or generation. Despite lack of apparent cumulative effect in the independent biomarkers, the combined single effects could together reduce overall cellular functioning and efficiency over time in wild birds exposed to pathogens, ozone, and anthropogenic food sources.

## Introduction

How organisms respond to environmental changes, such as urbanization, remains a pressing question, particularly during times of unprecedented anthropogenic impacts. On the one hand, urban environments may present novel resources for exploitation, such as new, predictable food sources (Jones and Reynolds, 2008; Oro et al., 2013). On the other hand, anthropogenic changes to the environment, like artificial light at night, air pollution, and anthropogenic noise, along with suboptimal nutrient composition in the diet are likely to challenge organismal health (Bonier, 2012; Isaksson, 2015; Ouyang et al., 2015; Isaksson and Bonier, 2020). Such challenges can be met with behavioral and physiological responses in order to avoid negative consequences (Tuomainen and Candolin, 2011; Sih, 2013; Watson et al., 2017; Ouyang et al., 2018). However, most responses have been evaluated when faced with only one stressor at a time and under controlled conditions. The potential responses to multiple stressors acting simultaneously are much less explored.

Oxidative stress, the imbalance between pro- and anti-oxidants, can occur either due to internally released pro-oxidants such as during high metabolic rate and oxidative bursts to defeat pathogens during an infection, or due to external sources such as inhalation of air pollutants (Isaksson, 2010; 2015). Repeated or chronically enhanced oxidative stress will lead to an accumulation of oxidative damage to biomolecules, which may ultimately cause premature death (Mudway et al., 2020). The oxidative stress status of urban-dwelling organisms has been investigated over the last decades, with a consensus that urban environments indeed lead to an increase in oxidative stress (Isaksson, 2010), but the magnitude of effect varies among studies (Isaksson et al., 2009; Rainio et al., 2013; Giraudeau and McGraw, 2014; Herrera-Duenas et al., 2014; de la Casa-Resino et al., 2015; Amri et al., 2017; Salmón et al., 2018b). Such context-dependency could be either caused by variation in pollutant types or concentrations, but it could also indicate that the response to pro-oxidants is modulated by other factors, such as the availability of important nutrients to support the antioxidant system (Isaksson, 2020) and the immune defense (Jiménez-Peñuela et al., 2022). However, the potential interplay between anthropogenic factors, infection, and nutrient availability remains largely unexplored (Jensen et al., 2023).

Diet quantity and quality are known to be influenced by urbanization (Seress et al., 2018; Jarrett et al., 2020). For some species, anthropogenic food sources may be beneficial as an additional, easy-accessible energy resource, and may even positively influence survival and performance in urbanized environments (Jones and Reynolds, 2008; Robb et al., 2008; Harrison et al., 2010). However, anthropogenic food sources as well as naturally occurring foods in the urban environment can be of poorer nutritional value (Isaksson and Andersson, 2007). Specifically, anthropogenic food often differs from naturally available food items in its composition of polyunsaturated fatty acids (PUFAs) (Simopoulos, 2002; Andersson et al., 2015). Indeed, urban birds have relatively lower circulating levels of ω3-PUFAs in their bloodstream than rural birds, especially during winter (Andersson et al., 2015; Isaksson et al., 2017). PUFAs are involved in several important physiological processes in animals, including, for example, growth during development (Twining et al., 2016b) and immune responses (Craig-Schmidt et al., 1987; Larsson et al., 2004). The short-chained ω6-PUFA linoleic acid (LA, 18:2n-6) and ω3-PUFA α-linolenic acid (ALA, 18:3n-3) are essential to birds and strictly acquired through diet (Larsson et al., 2004; Twining et al., 2021a). From these PUFAs, longer-chained PUFAs can be biosynthesized, although the efficiency of this process seems to vary between bird species (Twining et al., 2017; Twining et al., 2021b). Thus, the dietary availability of both short- and long-chained PUFAs can be imperative for physiological processes.

Immune responses are part of these processes since twenty-carbon PUFAs, like arachidonic acid (AA, 20:4n-6, a ω6-PUFA) and eicosapentaenoic acid (EPA, 20:5n-3, a ω3-PUFA), are pre-cursors to eicosanoids (i.e., prostaglandins, thromboxanes, leukotrienes), which function as immunomodulators. Importantly, eicosanoids show opposing inflammatory effects, depending on which substrate they originate from. Eicosanoids derived from ω6-PUFAs (AA) are considered mostly pro-inflammatory and to increase oxidative stress, while eicosanoids derived from ω3-PUFAs (EPA) exhibit anti-inflammatory properties and have been found to lower the production of ω6-derived eicosanoids by displacement or competitive inhibition (Craig-Schmidt et al., 1987; Tapiero et al., 2002; Larsson et al., 2004). Thus, the dietary balance between ω6-and ω3-PUFAs (i.e., the ω6:ω3-ratio) is critical for which pathway of eicosanoid production is favored (Fritsche et al., 1991) with subsequent health effects (Twining et al., 2016a). Subsequently, a diet that is relatively low in ω3-to ω6-PUFAs, which is likely in an urban environment (Andersson et al., 2015; Isaksson et al., 2017), may lead to a physiological state prone to inflammation or elicit increased oxidative damage due to a prolongation of the inflammatory response (Innes and Calder, 2018). The risk of oxidative stress may be further exacerbated by the increased exposure to air pollutants in cities, but research investigating the modulatory potentials of dietary fatty acids in response to air pollution is still limited (Whyand et al., 2018). Studies in humans have shown that a higher intake of ω6-PUFAs increases the adverse health effects of particulate matter (PM), while a higher intake of ω3-PUFAs reduces symptoms and severity of PM-induced health effects (Romieu et al., 2005; Brigham et al., 2019). Rodents and cell cultures of rodents that have been treated with ω3-PUFAs showed ameliorated responses to PM (Li et al., 2019) and ozone (Snow et al., 2018). In summary, the risk of oxidative stress through air pollution could be enhanced by a diet of anthropogenic food due to its lower content of ω3-PUFAs or be potentiated due to the pro-inflammatory properties of ω6-PUFAs. Investigating the potential interactions between air pollution, diet, and immune response on the avian oxidative status may therefore give mechanistic insights into the effects of anthropogenic environments on wildlife health.

Here, we performed a full-factorial laboratory experiment to investigate if different dietary composition of ω6-and ω3-PUFAs (i.e., ω6-*versus* ω3-rich diets) modulate the oxidative stress response towards pro-oxidative stimuli: ozone (*versus* normal air) and a simulated pathogen attack (lipopolysaccharide [LPS] *versus* control injections), using captive zebra finches (*Taeniopygia guttata*). We chose ozone as the air pollutant in this experiment since ground-level (tropospheric) ozone is a major component of urban air pollution and avian abundance and population growth rates have been shown to be negatively related to concentrations of tropospheric ozone levels, especially in mountain regions (Liang et al., 2020; Reif et al., 2023). Ozone originates mainly from chemical reactions of emission components of motorized vehicle combustion engines triggered by sunlight (Monks et al., 2015; Zhang et al., 2019). Over the last few years, background ozone levels have increased in urban areas compared to rural areas (Paoletti et al., 2014; Sicard et al., 2020). The effects of ozone exposure are wide-ranging, for example, causing severe health impairments, such as respiratory distress and disease through inflammation and immune activation, both in humans and birds (Rombout et al., 1991; Monks et al., 2015; Sanderfoot and Holloway, 2017; Fleming et al., 2018). The toxicity of ozone stems from its ability to either directly oxidize other molecules or to indirectly create free radicals and form reactive intermediate molecules which lead to further cascading oxidizing reactions (Mustafa, 1990).

Furthermore, injection with LPS simulates a pathogen attack, which triggers a costly acute-phase immune response (Owen-Ashley et al., 2006) and thereby, increases the release of pro-oxidants. To assess the oxidative stress response from the treatments, we measured concentrations of hydroperoxides in plasma as a proxy for overall pro-oxidant exposure and oxidative damage (using dROM assay). To get a broad estimate of the antioxidant defenses, we measured the circulating overall antioxidant capacity in plasma (OXY), as well as the intra-cellular and endogenously synthesized antioxidant glutathione in red blood cells, both the total levels (tGSH) and the oxidized form (GSSG). From these two measurements, the reduced (active form, GSH) can be calculated and the GSH/GSSG ratio can be used to infer the cellular antioxidant potential and their oxidative stress state.

Three main predictions can be outlined. First, we predicted that under elevated ozone levels, birds on a ω6-rich diet will have higher oxidative damage and/or a higher level of antioxidant defense than birds on a ω3-enriched diet. Second, we predict that birds on the ω6-enriched diet will have a stronger reaction when immune challenged, compared to birds on an ω3-enriched diet. This will be reflected in either higher levels of oxidative damage and/or upregulation of the antioxidant defense systems compared to birds on an ω3-enriched diet. Third, we predicted that the combined challenge of elevated ozone levels and an immune challenge would exacerbate the diets’ effects on oxidative damage and/or on the antioxidant defense system.

## Methods

### Experimental birds and general procedure

The experiment included a total of 189 adult male and female zebra finches and was carried out during January and February 2020 (see Supplementary Table S1 for more details about the sample sizes). Zebra finches were used as our model species since 1) we have a captive breed population at the Department of Biology of known age and life-history of the individuals, 2) they are not pre-exposed to any significant level of air pollution, 3) they can be kept in groups in cages, and 4) physiological assays has been optimized for the species. We divided the experimental birds into four batches with 47–48 individuals in each; two of the batches were exposed to clean normal air and two to elevated levels of ozone (detailed below, and in Supplementary Table S2). The birds forming a batch were caught weekly from the outdoor aviaries and brought inside the animal facility of the Department of Biology, Lund University, and kept in same-sex subgroups of six birds per cage (eight cages 97 × 52 × 83 cm). Prior to exposure, the eight subgroups of each batch were kept in two climate-controlled rooms at 24.4 ± 0.2 and 24.4°C ± 0.2°C (mean ± SD) and 49.5% ± 3.7% and 49.6% ± 3.8% relative humidity, respectively. The dark:light cycle was kept at 8 h:16 h for both rooms. Throughout the experiment, food (seed mixture: containing mainly millet seeds (around 85% yellow, white, red, and Japanese), and the rest consist of canary, niger, yellow, and red panicum seeds; Exoten, Benelux, Kinlys Group, Belgium) and water were provided *ad libitum*, with an additional fresh slice of cucumber each day and each cage contained a cuttlebone for calcium supplementation. Body mass was measured with a digital scale to the nearest 0.1 g (i.e., body mass at the start of the experiment). The birds were kept under these conditions for 5 days for acclimatization. On day six, we started the two diet treatments (described below). On day 12, we transferred the birds to the Aerosol laboratory at the Department of Design Sciences, Lund University, where they remained in the same cage groups. The birds were housed in cages, which were standing in a stainless-steel exposure chamber that measured 22 m^3^ and had a floor area of 9 m^2^ (Isaxon et al., 2013). The chamber was continuously ventilated with clean normal air from outside the chamber, −44 m^3^/h, resulting in an exchange rate of the air in the chamber of 2 times per hour. The air supply for the chamber was passed through an activated carbon filter and an ultra-low penetration air filter, removing both gas phase and particle air pollutants, while an air conditioning unit maintained a constant temperature and relative humidity of 23.2°C ± 1.4°C and 30.3% ± 3.1% (mean ± SD), respectively. The chamber was illuminated by LED panels (4000 K), that were placed 20 cm above the cages and provided light from 5:00 to 21:00 at 3200 ± 690 lx (mean ± SD). The birds continued receiving food according to their diet treatment (see below). For the batches exposed to ozone, the ozone exposure (described below) started on day 12, immediately after the birds were brought into the exposure chamber. The exposure lasted for 5 days until day 17. In the early morning of day 17, half of the birds of each batch were subjected to an immune challenge (see description below), while the other half served as a control group and received an injection of phosphate-buffered saline (PBS; see description below). In the evening of the fifth exposure day and when the experiment ended, a blood sample was collected from every bird (see below) and body mass was measured with a digital scale to the nearest 0.1 g (i.e., post-exposure body mass; due to human error one bird is missing this measurement). After the experiment, all birds were brought back to the outdoor aviaries.

### Diet treatment

The diet treatment started on day six (7 days before the exposure started), to allow for a longer time to integrate the PUFAs into tissues and plasma. We split each batch of birds into half, so that one-half received a diet with an elevated ω6-PUFA intake, while the other half was fed a diet with increased levels of long-chained ω3-PUFAs. Each subgroup (cage) of six birds always received the same diet type. Hence, per batch, four subgroups received a high ω6:ω3 ratio diet (henceforth called ω6-rich diet) and four subgroups received a low ω6:ω3 ratio diet (henceforth called ω3-rich diet). Both diets were freshly prepared each morning and consisted of a 33.3:1 (w/w) of seeds (Exoten, Benelux, Kinlys Group, Belgium) and the specific ω6-or ω3-rich oil. Additionally, we added potato starch in a 1:10 ratio (v/w) to obtain the natural appearance of the seeds. We used algae oil (Omega-3 Algolja, Holistic Sweden AB) for the ω3-rich diet, since the oil contains EPA (21%) and docosahexaenoic acid (DHA, 22:6n3; 21%), in addition to containing 48% palmitic acid (saturated fatty acid; SFA) and 10% oleic acid (monounsaturated fatty acid; MUFA). The oil used for the ω6-rich diet was a 3:1 (w/w) mixture of sunflower seed oil (Solrosolja kallpressad, Kung Markatta, Midsona Sverige AB) and coconut oil (Ekologiska Kokosolja kallpressad virgin, ICA AB). The latter was added to achieve similar levels of SFA as the ω3-rich diet oil. The main fatty acid in the ω6-rich diet oil was linoleic acid (LA). We analyzed the plasma fatty acid concentrations of a subset of individuals (N_tot_ = 23 females, i.e., *n* = 5–6 per diet and exposure group) at the end of the experiment to confirm the effectiveness of the two diet treatments (details in Supplementary Table S3). As predicted, birds on a ω3-rich diet had higher concentrations of total ω3-PUFAs (*p* < 0.001), lower total ω6-PUFA concentration (*p* = 0.02), a lower ω6:ω3 ratio (*p* < 0.001), lower LA and AA concentrations (*p* = 0.04 and *p* < 0.001, respectively), and higher ALA, DHA, and EPA concentrations (*p* = 0.03, *p* < 0.001 and *p* < 0.001, respectively) compared to the birds on the ω6-rich diet. A comparison to fatty acid profiles of birds from the same population fed the same seed mixture, but without the oil manipulations, indicates a 3-fold change from baseline in ω6:ω3 PUFA ratios (in both directions) as a result of the diet manipulations in the present study (Egger-Peitler, 2017; unpublished MSc thesis).

### Ozone exposure

Birds were either exposed to elevated levels of ozone or to normal air, following an alternating weekly schedule, starting with exposure to normal air (batch 1) followed by ozone exposure (batch 2), and so forth for the four batches. Ozone was constantly generated by a modified commercially available spark discharge generator (Model AM 3000–2, Ozone Technology AB, Sweden). The ozone generation was introduced into the air going into the chamber and regulated by an on-off switch through a feedback loop from an ozone analyzer (Model 49i, Thermo Fisher Scientific, United States of America) placed in the chamber, set to keep to ozone level at 100 ppb. The ozone concentration in the chamber was 109 ± 0.05 ppb (mean ± SE) during the ozone exposure (i.e., weeks 2 and 4) and 3.7 ± 0.01 ppb for the normal air exposure (i.e., weeks 1 and 3).

An example of current national air quality standards is that of the United States (NAAQS, U.S. EPA 2021), where the 8 h average ozone concentration (averaged over 3 years) should not exceed 70 ppb. The corresponding 8 h average is for China set at 80 ppb (Monks et al., 2015) and for the EU the 8 h average should not exceed 60 ppb for more than 25 days per year, averaged over 3 years (European Environment Agency, 2020). Despite nationwide efforts to decrease ozone levels, about 41% of all reporting stations for the European air monitoring reported concentrations above target values in 2018 (European Environment Agency, 2020) and equally 41% of the stations exceeded legislation thresholds for ozone concentrations in the US in 2014 (Paoletti et al., 2014). In humans, an increase of 10 ppb in ozone concentration increases the risk of death from respiratory causes by 4% (Jerrett et al., 2009).

### Immune challenge

We applied the immune challenge treatment on the fifth day of exposure (i.e., on day 17 from the start of the experiment), for both the ozone and normal air treatment, respectively, starting at 6a.m. We used LPS (lipopolysaccharide from *Escherichia coli* 055: B5, Sigma Aldrich, Sweden), which is an endotoxin on the cell wall of Gram-negative bacteria and is commonly used to induce an innate immune response. Within each batch and diet treatment, we split the birds into two additional groups. Half were subjected to an immune challenge by subcutaneous injections of LPS, with the dosage of 1 µg LPS diluted in 2.2 µL of phosphate-saline buffer (PBS) per Gram of body mass. The other half received a subcutaneous injection of only PBS (a similar dosage of 2.2 µL PBS g^−1^). 11.8 ± 0.6 h (mean ± SD) later we drew a blood sample (∼90 µL) from the jugular vein. This time interval was set to capture the time window where most physiological changes occur in response to LPS (e.g., Hegemann et al., 2013). The blood sample was centrifuged for 10 min at 4°C at 6 500 rpm, the plasma was then separated from the red blood cells (RBCs), and both were snap-frozen in liquid nitrogen. All samples were stored at −80°C until analysis.

### Assays for oxidative status

#### Oxidative defense

We quantified the non-enzymatic antioxidant capacity using the OXY-adsorbent assay (Diacron International, Grosseto, Italy). This assay measures the capacity of antioxidant components in the plasma to cope with a pro-oxidant attack through hypochlorous acid (HOCl). In short, plasma was 1:100 diluted with double-distilled water. Two microliters of the dilution were mixed with a HOCl solution in a microplate and incubated at 37°C for 10 min while shaking at 500 rpm. Then, 2 μL of chromogen (*N,N*-diethyl-p-phenylenediamine) were added to each well and the absorbance at 490 nm was immediately measured in a microplate reader (FLUOstar OMEGA, BMG LABTECH). Concentrations are expressed as millimoles of neutralized HOCl and calculated with a 4-point standard curve (340–42.5 mM HOCl neutralized). All samples, including standards and control samples, were run in duplicates and samples were randomized over plates. Due to the time sensitivity of the assay, we divided each plate into three separate runs, each having a standard curve and a control sample. The inter-assay and mean coefficient of variation between duplicates were: 13.7% (calculated from means of three controls per plate) and 2.5%, respectively.

We measured the concentration of total glutathione (tGSH) and the oxidized form of glutathione (GSSG) in RBCs, following Baker et al., 1990; Isaksson et al., 2013 adapted to a microplate reader. Glutathione is one of the most abundant and important cellular antioxidants, which not only directly scavenges ROS, but also functions as a cofactor for enzymatic antioxidants (Meister and Anderson, 1983). The ratio of the reduced (GSH) to the oxidized (GSSG) form of glutathione (GSH/GSSG) is commonly used as a marker of current oxidative stress, with lower ratios indicating a shift towards higher concentrations of the oxidized form of glutathione. We calculated the GSH concentration by subtracting the GSSG concentration from the tGSH. Briefly, we first diluted 8 mg of RBCs in phosphate-buffered saline 1:1 (w/v). Then, 8 µL of the dilution was mixed with 16 µL of 5% SSA (5-sulfosalicylic acid) and left incubating on ice for 10 min. Afterwards, the samples were centrifuged at 4°C and 10,000 rpm for 10 min. We diluted 10 µL of the hemolysate supernatant with 390 µL of GSH-buffer (143 mM NaH_2_PO_4_, 6.3 mM EDTA, pH 7.4). Twenty microliters were transferred to a flat-bottom microplate and 170 µL of reaction solution (10 mmol L^−1^ 5,5′-dithiobis 2-nitrobenzoic acid and 0.34 U of glutathione reductase in GSH buffer) were added. The reaction was started by adding 34 µL of 2 mM NADPH (nicotinamide adenine dinucleotide phosphate, in GSH-buffer). After shaking the plate, absorbance was measured at 412 nm (FLUOstar OMEGA, BMG LABTECH) every 30 s over 5 min. Concentrations are expressed in μmol l^−1^ and calculated with a 6-point standard curve, ranging from 100 μM to 3.12 µM. The samples were run in duplicates, and randomized over plates, with an intra-assay coefficient of variation of 2.8% and an inter-assay coefficient of variation of 14.3%.

For the quantification of the GSSG concentration, we treated 200 µL of the diluted hemolysate supernatant with 5 µL of a 2-vinylpyridine solution (1:4, 2-vinylpyridine and 99% EtOH) and incubated it for 1 h at room temperature while shaking at 1400 rpm. After incubation, the GSSG sample was centrifuged for 5 min at 12,000 rpm at 4°C. Then, the microplate was loaded and treated in the same manner as for tGSH, with the exception that the reaction solution contained only 0.17 U of glutathione reductase. For GSSG, we also ran a serial dilution standard curve with six points on each plate, ranging from 10 μM to 0.313 µM. The samples were run in duplicates, and randomized over plates, with an intra-assay coefficient of variation of 3.6% and an inter-assay coefficient of variation of 18.9%.

#### Oxidative damage

We quantified oxidative damage using the dROM assay (Diacron International, Grosetto, Italy). This assay measures the concentration of hydroperoxides in the plasma, which are oxidative damage compounds generated through oxidative damage to lipids, proteins, and nucleic acids. In short, 4 µL of plasma was diluted with 200 µL of acidic buffer solution (containing acetate buffer, pH 4.8, and an aromatic alkyl-amine as a chromogen in a 100:1 ratio). Then, the samples were incubated for 75 min at 37°C. Absorbance was measured at 490 nm after incubation with a microplate reader (FLUOstar OMEGA, BMG LABTECH). The ROMs concentration was calculated with a 6-point standard curve and is expressed in mM H_2_O_2_ equivalents. Samples, including standard curve and control samples, were run in duplicates, and randomized over plates, with an intra-assay coefficient of variation of 5.2% and an inter-assay coefficient of variation of 4.3%.

### Statistical analyses

All statistical analyses were performed in R 4.1.0 (R CoreTeam, 2020). Detailed sample sizes for each model can be found in S1 of the Supplementary Material. The effects of diet, ozone exposure, and immune challenge on different oxidative stress status markers were tested with linear mixed models (LMMs), using the package “lmerTest” (Kuznetsova et al., 2017). Full models included diet treatment (ω6-rich or ω3-rich diet), ozone treatment (ozone exposure or normal air), and immune challenge (LPS or PBS injection) as fixed factors. The full model also included all two-way and three-way interactions between diet, ozone treatment, and immune challenge. Mean-centered post-exposure body mass was included as a covariate in the models for the oxidative stress status markers. As additional fixed effects, we included sex (male/female) and replicate (weeks 1 and 2 *versus* weeks 3 and 4), to account for the replication of the experiment. As random effects, all models included the assay plate number (where applicable) and the cage number to account for potential differences between cages or their positions within the exposure chamber. However, in the models for OXY and the GSH/GSSG ratio, we excluded cage number as a random effect since its variance was estimated to zero. Normality of residuals was visually inspected, and ROMs levels were log-transformed to achieve normal distribution. We identified and excluded four individuals in the GSH/GSSG ratio model as outliers (a male and a female on the ω6-rich diet/normal air/LPS injection; a male on the ω3-rich diet/normal air/LPS; and one female on ω6-rich diet/normal air/PBS), using a Rosner’s generalized extreme Studentized deviate test from the package “EnvStats” (Millard, 2013). We performed stepwise-backward model reductions of non-significant (*p* > 0.05) terms, with the treatments (diet treatment, ozone treatment, and immune challenge), always being retained in the final model. Significant interaction terms were followed up by *post hoc* pairwise comparisons, using the package “emmeans” (Lenth et al., 2020). Denominator degrees of freedom were calculated using Satterthwaite approximation and estimates were calculated using restricted maximum likelihood. Statistics of dropped terms were calculated using maximum likelihood and can be found in Supplementary Table S4. Presented means and standard errors (SE) are calculated from the predicted values of final models.

## Results

### tGSH

**FIGURE 1:**
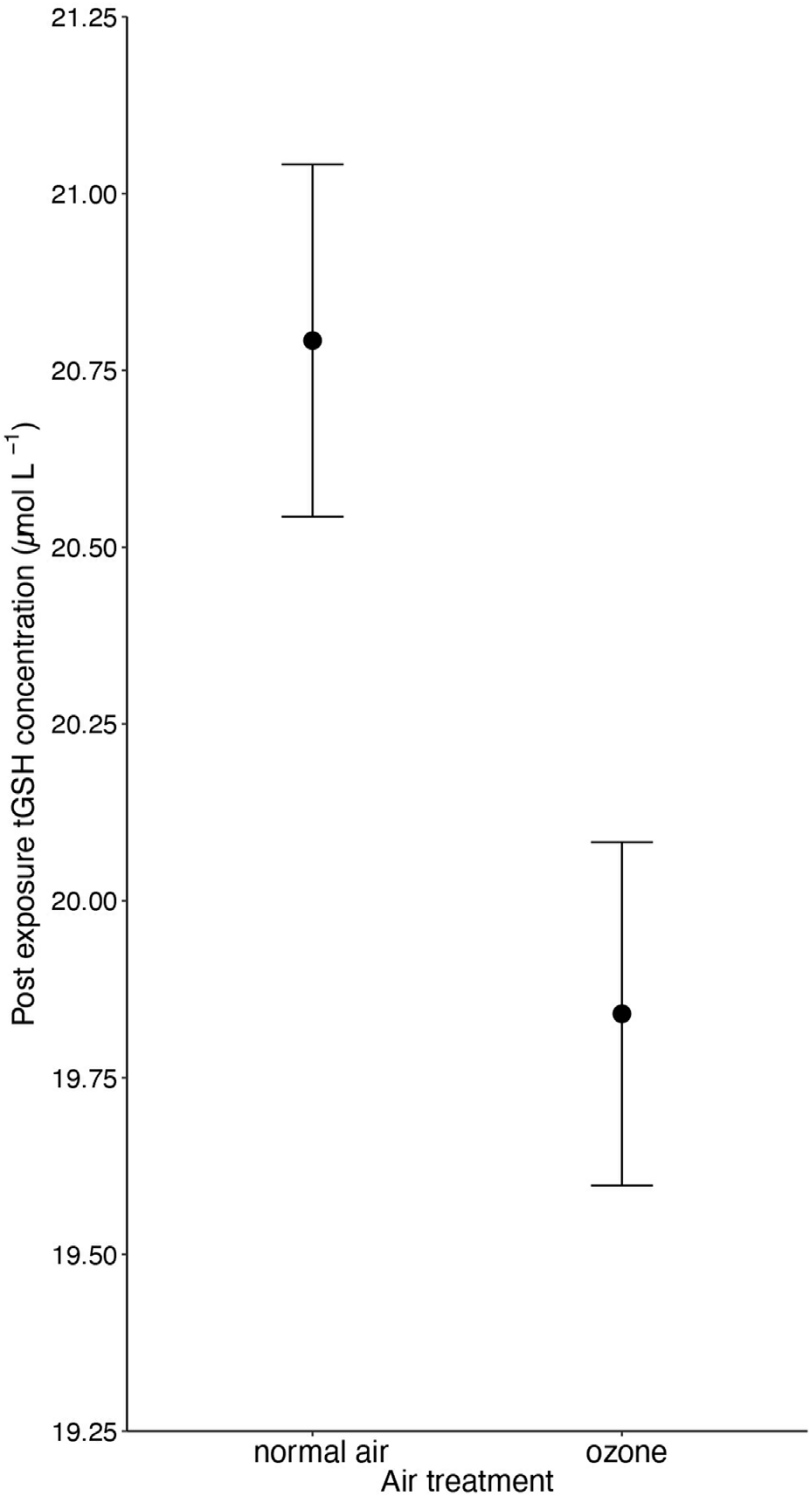
The negative effect (*p* = 0.017) of 5-day ozone exposure on total glutathione levels (tGSH) in red blood cells of male and female zebra finches (*Taeniopygia guttata*). Means ± standard errors of predicted values from final models are shown (n_air_ = 88 and n_ozone_ = 91).

Ozone-exposed birds showed significantly lower tGSH levels (19.84 ± 0.24 µM; mean ± SE from predicted values) compared to birds exposed to normal air (20.79 ± 0.25 µM; 4.6% difference between the treatments, F_1, 160.203_ = 5.77, *p* = 0.017; Figure 1 and Table 1). Body mass at the end of the experiment was positively associated with tGSH levels (F_1, 165.516_ = 17.68, *p* < 0.001, Table 1). No other single treatment, nor the interactions between treatments influenced tGSH levels (all *p* > 0.19, Table 1; Supplementary Table S4).

**TABLE 1:** Output of final linear mixed models, using captive zebra finches (*Taeniopygia guttata*) of both sexes, testing responses of concentration of total glutathione (tGSH), ratio of reduced/oxidized glutathione (GSH/GSSG), non-enzymatic antioxidant capacity (OXY), and oxidative damage levels (ROMs) after a 5 day experimental exposure to either ozone or normal air (air treatment), while feeding on either of the two different fatty acid diets (ω6-or ω3-rich) and subsequent immune challenge (phosphate-buffered saline [PBS] injection or lipopolysaccharide [LPS] injection). *p*-values <0.05 are in bold. SE = standard error; df = degrees of freedom; StDev = Standard deviation; SS = sum of squares.

tGSH concentration					
Random effects	Variance	StDev	# Groups	# Observations	
Plate	4.698	2.168	11	179	
Experimental cage	0.090	0.300	8		
Residual	8.651	2.941			
**Fixed effects**	**Estimate ± SE**	**SS**	**df**	**F**	**P**
Intercept	20.767 ± 0.808				
**Centred body mass**	**0.525 ± 0.125**	**152.916**	**1, 165.516**	**17.68**	**< 0.001**
Diet (ω3)	−0.606 ± 0.456	15.253	1, 161.430	1.76	0.19
Immune challenge (LPS)	0.467 ± 0.444	9.536	1, 159.447	1.10	0.30
**Air treatment (ozone)**	**−1.084 ± 0.451**	**49.932**	**1, 160.203**	**5.77**	**0.017**

GSH/GSSG ratio					
Random effects	Variance	StDev	# Groups	# Observations	
Plate	0.563	0.750	12	172	
Residual	0.550	0.742			
**Fixed effects**	**Estimate ± SE**	**SS**	**df**	**F**	**P**
Intercept	3.447 ± 0.253				
Diet (ω3)	−0.299 ± 0.168	0.153	1, 157.93	0.28	0.60
Immune challenge (LPS)	0.149 ± 0.114	0.931	1, 156.37	1.69	0.20
Air treatment (ozone)	−0.144 ± 0.163	0.339	1, 157.09	0.62	0.43
**Diet x air treatment**	**0.472 ± 0.231**	**2.288**	**1, 156.84**	**4.16**	**0.043**

Non-enzymatic antioxidant capacity (OXY)					
Random effects	Variance	StDev	# Groups	# Observations	
Plate	434.4	20.84	15	172	
Residual	1951.6	44.18			
**Fixed effects**	**Estimate ± SE**	**SS**	**df**	**F**	**P**
Intercept	225.201 ± 8.877				
**Centred body mass**	**4.928 ± 1.950**	**12,461.7**	**1, 159.29**	**6.39**	**0.012**
**Diet (**ω**3)**	**−14.400 ± 6.904**	**8489.6**	**1, 157.89**	**4.35**	**0.039**
Immune challenge (LPS)	2.429 ± 6.872	243.9	1, 157.03	0.13	0.72
Air treatment (ozone)	−7.987 ± 6.987	2550.0	1, 160.78	1.31	0.26

Oxidative damage (ROMs; log-transformed)					
Random effects	Variance	StDev	# Groups	# Observations	
Plate	0.003	0.057	9	155	
Experimental cage	0.001	0.037	8		
Residual	0.096	0.310			
**Fixed effects**	**(Log) Estimate ± SE**	**SS**	**df**	**F**	**P**
Intercept	0.284 ± 0.062				
**Centred body mass**	**0.095 ± 0.014**	**4.510**	**1, 145.40**	**47.08**	**< 0.001**
**Sex (male)**	**0.197 ± 0.051**	**1.450**	**1, 144.50**	**15.13**	**< 0.001**
Diet (ω3)	−0.048 ± 0.051	0.086	1, 143.78	0.90	0.35
**Immune challenge (LPS)**	**0.302 ± 0.050**	**3.475**	**1. 140.97**	**36.28**	**< 0.001**
Air treatment (ozone)	−0.018 ± 0.051	0.013	1, 144.58	0.13	0.72

### GSH/GSSG ratio

**FIGURE 2:**
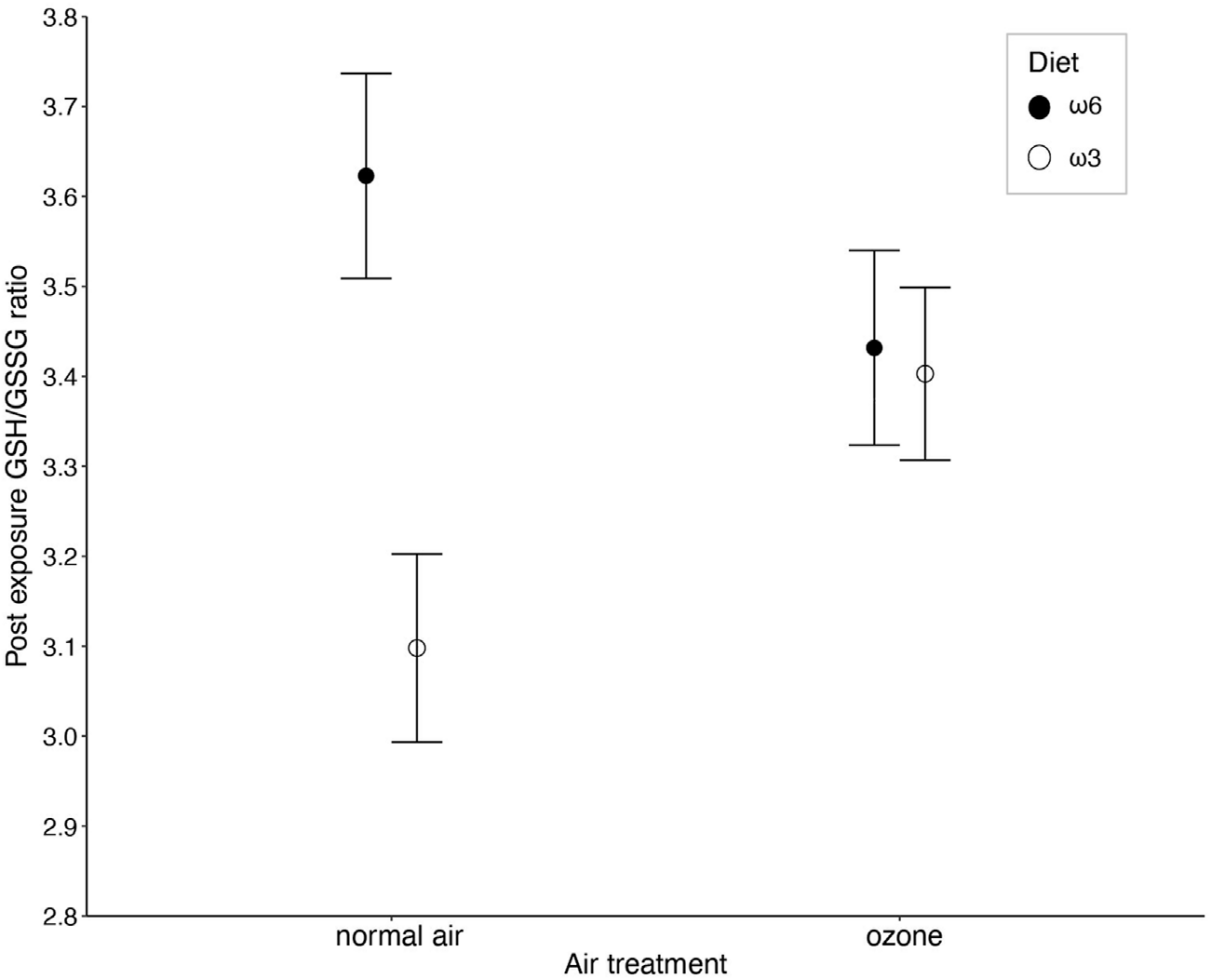
The interaction between ozone and diet treatment (i.e., ω6-and ω3-rich diets, *p* = 0.043) on the ratio between reduced (GSH) and oxidized (GSSG) glutathione (GSH/GSSG ratio) measured in red blood cells of male and female zebra finches (*Taeniopygia guttata*), after 5 days of exposure. Means ± standard errors of predicted values from final models are shown (n_air+ω6_ = 43, n_air+ω3_ = 40, n_ozone+ω6_ = 45, and n_air+ω3_ = 44).

The ratio of reduced over oxidized glutathione was different between the diet treatments, but the effect was dependent on whether birds were exposed to ozone or not (i.e., diet × ozone treatment: F_1, 156.84_ = 4.16, *p* = 0.043; Figure 2 and Table 1). Specifically, under normal air conditions, a diet rich in ω3-PUFAs led to a lower GSH/GSSG ratio (3.04 ± 0.59) compared to a ω6-rich diet (3.62 ± 0.74), suggesting a lower antioxidant potential of ω3-PUFA birds within their red blood cells. The difference between the diet group disappears under elevated ozone conditions (ω3-rich diet: 3.40 ± 0.63, ω6-rich diet: 3.43 ± 0.72, Figure 2). However, *post hoc* pairwise comparisons did not indicate any significant differences between groups (all *p* > 0.2). No other interaction terms were significant (all *p* > 0.14, Supplementary Table S4). Neither immune challenge, nor sex, replicate or body mass influenced the GSH/GSSG ratio (all *p* > 0.12, Table 1; Supplementary Table S4).

### Antioxidant capacity—OXY

**FIGURE 3:**
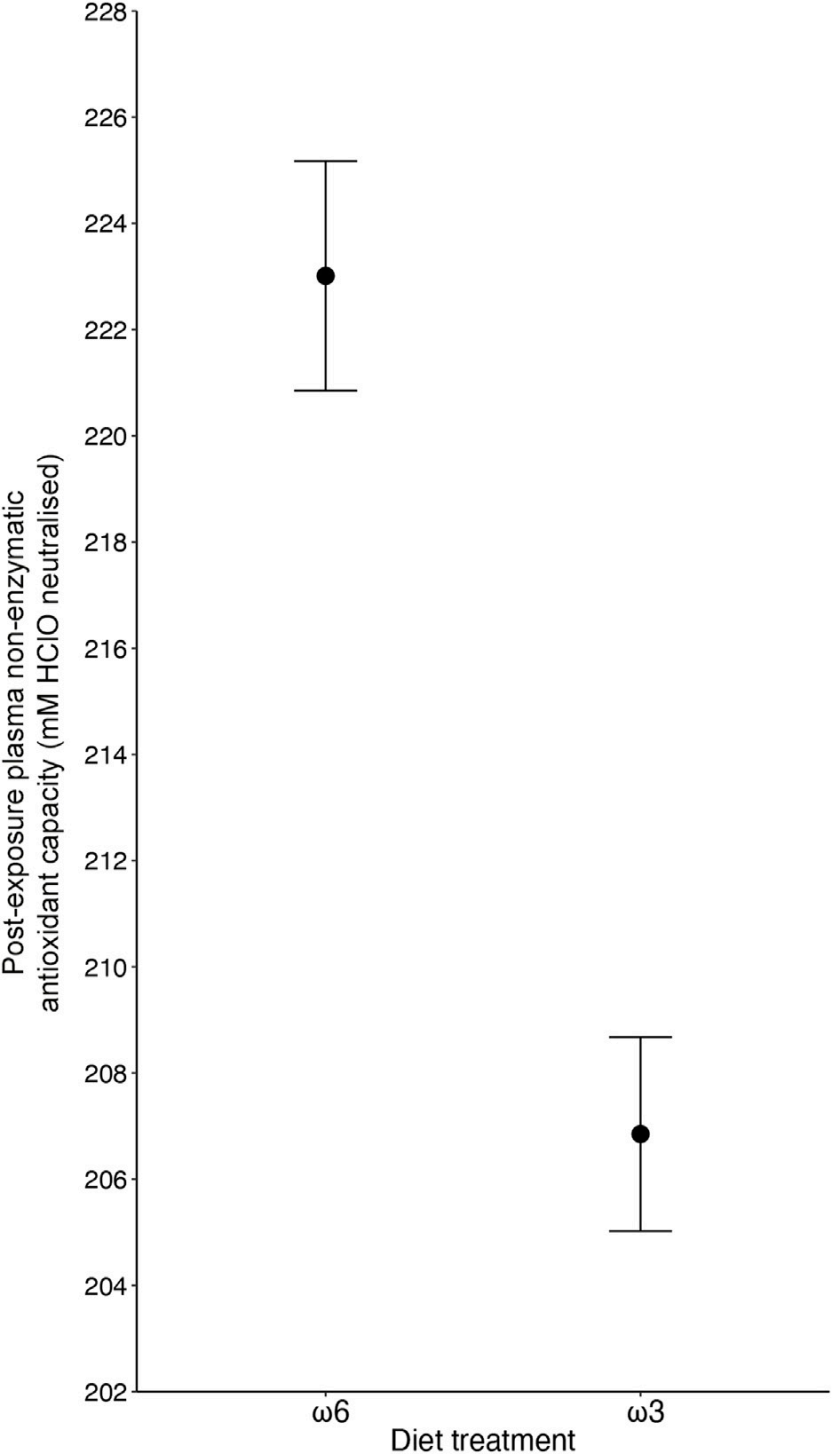
The effect of diet treatment (i.e., ω6-and ω3-rich diets, *p* = 0.039) on plasma non-enzymatic capacity (OXY) levels of male and female zebra finches (*Taeniopygia guttata*), with ω3-fed birds showing lower levels after 5 days of exposure. Means ± standard errors of predicted values from final models are shown (n_ω6_ = 87, and n_ω3_ = 85).

Plasma antioxidant capacity was influenced by the specific PUFA diets the birds received, with birds on a ω3-rich diet having a 7.8% lower capacity (206.85 ± 1.83 mM HClO neutralized) compared to birds on a ω6-rich diet (223.01 ± 2.16 mM HClO neutralized) (F_1, 157.03_ = 4.35, *p* = 0.039; Figure 3 and Table 1). Like tGSH, body mass measured at the end of the exposure was positively correlated with OXY levels (F_1, 159.29_ = 6.385, *p* = 0.012, Table 1). None of the interaction effects between the different treatments, nor immune challenge, ozone/normal air treatment, sex, or replicate, predicted OXY levels (all *p* > 0.35, Table 1 and Supplementary Table S3).

### Oxidative damage (ROMs)

**FIGURE 4:**
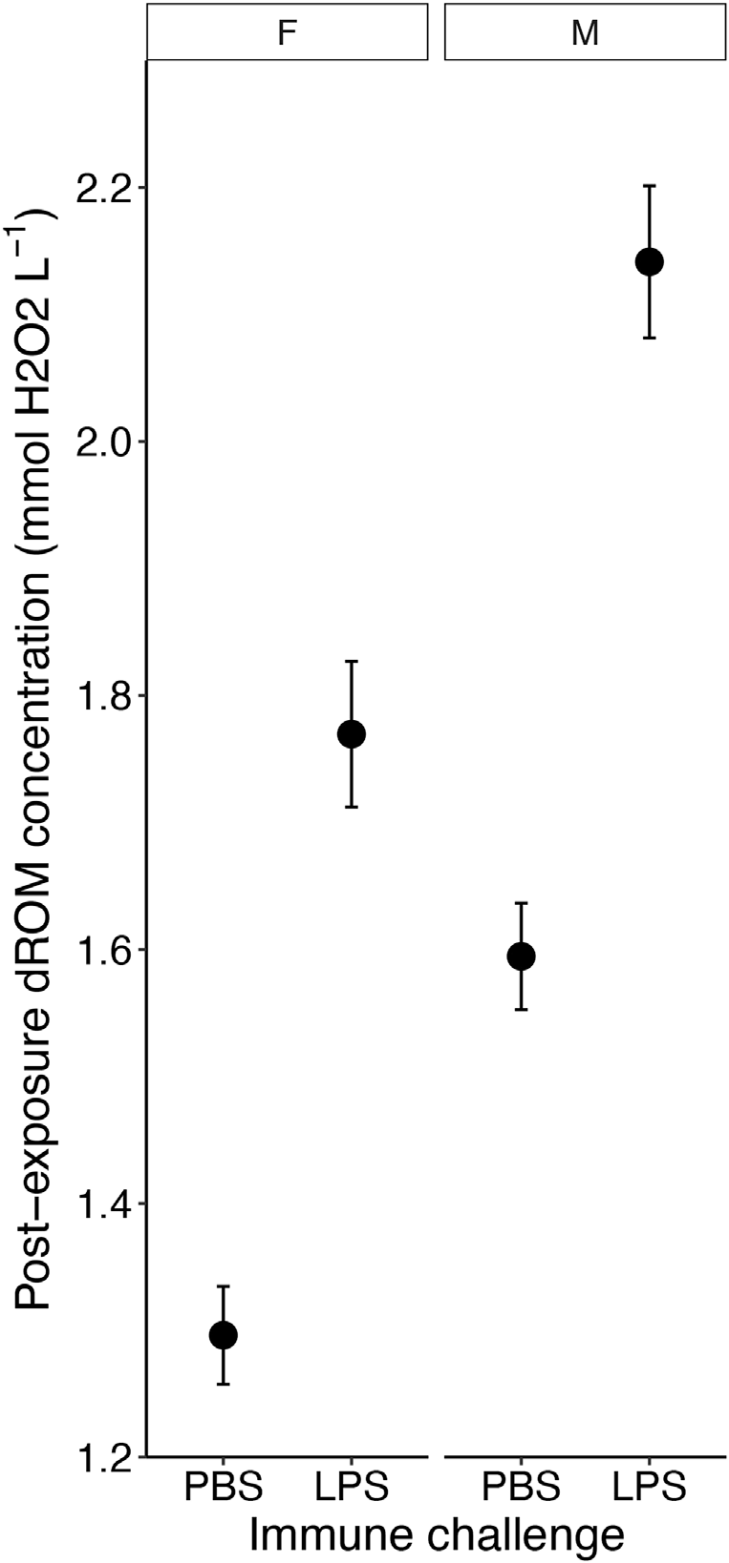
The effect of the immune challenge—lipopolysaccharide [LPS] injection *versus* controls (phosphate-buffered saline [PBS] injection) on the concentration of reactive oxygen metabolites (ROM) on the fifth day of exposure of male and female zebra finches (*Taeniopygia guttata*). Means ± standard errors of predicted values from final models are shown (n_PBSfemale_ = 36, n_PBSmale_ = 39, n_LPSfemale_ = 36, n_LPSmale_ = 44).

Levels of oxidative damage at the end of the experiment, measured as ROMs concentrations, were 26.5% higher (1.97 ± 0.05 mM H_2_O_2_ equivalents) in birds that received an immune challenge compared to birds that received a PBS injection (1.45 ± 0.33 mM H_2_O_2_ equivalents) (F_1, 140.97_ = 36.28, *p* < 0.001; Figure 4 and Table 1). Furthermore, males had higher ROMs levels (1.88 ± 0.05 mM H_2_O_2_ equivalents) at the end of the experiment than females (1.53 ± 0.04 mM H_2_O_2_ equivalents) (F_1, 144.50_ = 15.13, *p* < 0.001; Table 1). Heavier birds at the end of the experiment also had higher levels of ROMs (F_1, 145.40_ = 47.08, *p* < 0.001, Table 1). We found no interaction effects of our different treatments on levels of oxidative damage (all *p* > 0.19; Supplementary Table S4). Diet and ozone treatment did not influence ROMs levels (both *p* > 0.34, Table 1), nor did replicates (*p* = 0.23, Supplementary Table S4).

### Body mass

The body mass at the end of the experiment was not affected by any of the applied treatments (all *p* > 0.6, see Supplementary Table S5). Body mass at the start of the experiment was different for the two replicates, with the birds in the first replicate (i.e., week 1 (normal air) and 2 (ozone)) being 0.71 g lighter than the birds in the second replicate (i.e., week 3 (normal air) and 4 (ozone)) (F_3_ = 8.11, *p* = 0.005). This mass difference remained stable during the experimental period, i.e., first replicate were 0.77 g lighter than the birds in the second replicate at the end of experiment (F_1,176.44_ = 8.76, *p* = 0.003). Males were 0.52 g heavier at the beginning of the experiment than females (F_1_ = 4.37, *p* = 0.038), but this difference disappeared at the end of the experiment (*p* = 0.30).

## Discussion

Urban wildlife needs to deal with several human-introduced factors, such as changed diet composition, traffic-related air pollutants, and changed fauna of pathogens. These factors do not act in isolation but can interact, and thereby either exacerbate or mitigate negative effects of living in urban environments. To investigate this, we performed a full-factorial experiment using captive zebra finches. The main aim was to explore the modulatory role of dietary PUFAs on the oxidative stress response to ozone and/or a simulated pathogen attack—two pro-oxidative agents. Our results show that both a diet differing in ω6:ω3 PUFA ratio, exposure to ozone, and an immune challenge have the capacity to alter the oxidative stress status of birds. However, the modulatory effect of diet was less pronounced than predicted and only one marker revealed such an effect.

The modulatory effect of dietary PUFAs was revealed for the ratio between GSH/GSSG, but only under normal air and not when the birds were exposed to ozone. More specifically, birds fed a diet rich in long-chained ω3-PUFAs had a lower GSH/GSSG ratio compared to birds fed ω6-PUFAs in normal air conditions. For context, it is important to note that tGSH (GSSG + GSH) did not differ between our two diet treatments, meaning the results were not driven by absolute differences in total glutathione. Traditionally, the ratio of GSH/GSSG has been interpreted as a marker of current oxidative stress (i.e., a lower ratio (more GSSG) indicates a higher level of oxidative stress), which would suggest that a ω3-rich diet has a negative impact on the oxidative state within the red blood cells, under normal air conditions, but that this effect decreases when exposed to ozone. Given that another study has shown that ω3-supplementation increases the rate of oxidation of GSH when exposed to ozone (Corteselli et al., 2020), we find it unlikely that the ω3-group here experienced higher oxidative stress in normal air than when exposed to the extremly potent pro-oxidant ozone, or compared to the ω6-birds. Our measurement does not take oxidation rate in account, just the absolute concentration of GSSG and GSH. However, to compensate and minimize the pro-oxidant attack, the glutathione system uses glutathione reductase (GR) for catalyzing GSSG back into its active form (i.e., GSH). The expression of GR is regulated by the presence of pro-oxidants, hence when GR is upregulated the GSH/GSSG ratio will increase (Smith et al., 1996; Schafer and Buettner, 2001; Kim et al., 2010). Possibly, this is the reason for the increased ratio in the ω3-birds when exposed to ozone. Furthermore, the continuous high ratio among the ω6-birds, regardless of exposed to ozone or not, might be related to the well-known pro-inflammatory and pro-oxidative effect of dietary ω6-PUFAs (Innes and Calder, 2018). The two-differential diet regimes started 7 days prior to exposure treatments, and 12 days prior to blood sampling, hence the lack of effect of ozone at day 12 in the ω6-birds might be due to an already upregulated GR in response to the ω6-diet seen in the normal air group. This interpretation is further corroborated by the higher plasma antioxidant activity (i.e., OXY) in the ω6-birds compared to the ω3-birds, independently of ozone treatment. It should be noted that, although our interpretation is physiologically and theoretically sound, we regrettably did not measure GR activity, thus the underlying mechanism behind the observed pattern is not fully clarified by our results.

Moreover, we found that ozone exposure alone decreased the cellular concentration of total glutathione. The glutathione redox system is one of the most abundant and important antioxidant defenses in an organism (Meister and Anderson, 1983). The present exposure level of 109 ± 0.05 ppb for 5 days indicates slow depletion of glutathione from the cellular antioxidant machinery. A reduction of glutathione concentration has important implications on organismal condition, as a depletion of glutathione has been found to lead to cell dysfunction and apoptosis (Franco and Cidlowski, 2009). While a decrease of about 5% over 5 days in the ozone-exposed birds may seem a modest reduction, this begs the question of the consequences of more long-term ozone exposure on avian health. In accordance with the present study, toxicological studies of European starlings (*Sturnus vulgaris*) also reveal a depletion of glutathione when exposed to a mix of urban air pollutants, both in the wild and in a controlled setting when exposed to vehicle exhaust (North et al., 2017a; 2017b). However, other correlative studies of urban birds and pollution levels reveal no effect on glutathione levels, but note that ozone levels were not measured in any of these studies (Salmón et al., 2018b; Li et al., 2021). Experimental studies of ozone specifically and its effects on antioxidants have primarily been done on mammals, either *in vitro* or *in vivo,* and instead of using biomarkers in the blood most of these studies focus on other fluids (such as epithelial lining fluid) or specialized cells in the respiratory system (e.g., Rietjens et al., 1985; Avissar et al., 2000). Hence, comparing studies is difficult due to widely varying tissues/fluids used, biomarkers, species but also due to wide ranges of ozone concentrations (1–800 ppb) and exposure times (4 h—1 week). However, given that blood has been proposed to be an insensitive tissue for ozone exposure (Kadiiska et al., 2011), the fact that we here see a 5% reduction in tGSH in red blood cells raises concern about the oxidative status of other tissues.

In addition to the pro-oxidative ozone exposure, we also immune-challenged half of the birds with an injection of LPS to trigger a release of ROS to combat the presumed infection. In contrast to the ozone, the pro-oxidants released during this oxidative burst is an adaptive mechanism to destroy pathogens. In line with this, the immune-challenged birds suffered from higher levels of oxidative damage. Since accumulation and/or re-occurring oxidative damages over time is directly linked to age-related loss in cellular function (Martin and Grotewiel, 2006) a continued oxidative burst could have a profound long-term consequence for the organism (Costantini and Møller, 2009). Yet, the immediate benefit of this adaptive response must overrides the long-term costs. Interestingly, and in line with this adaptive response, the antioxidant system is not triggered to defeat this internal production of ROS, since neither the antioxidant capacity in the plasma (OXY) nor the antioxidant markers in the red blood cells (tGSH and GSH/GSSG) were upregulated. Increased oxidative stress and subsequent oxidative damage have been shown previously after an immune response to fight invading pathogens (Isaksson et al., 2013; Armour et al., 2020). However, our study suggests that this increased oxidative stress is decoupled from the different antioxidant potential of PUFA intake, as well as additional pro-oxidant exposure via ozone; we found no indication that the effects of oxidative burst were modulated by these two treatments. This could indicate a mechanism where the oxidative burst to defeat pathogens overrides the potentially protective effects of diet and detoxification of pro-oxidants.

Lastly, we found less evidence for cumulative and/or modulatory effects between the three experimental treatments—differential PUFA-diet, ozone exposure, and immune challenge—than expected. It has previously been proposed that the magnifying effect of multiple anthropogenic factors might be overstated since interactions often are assumed to be synergistic (Côté et al., 2016). However, although we were neither able to show cumulative nor synergistic effects of the combined treatments in independent biomarkers, the overall effect on the physiological state when taking all effects into account could potentially worsen the condition of wild urban birds. For example, an increased accumulation of oxidative damage (e.g., after an infection) could be more detrimental to the urban than rural birds, given that they also can have the challenge of a reduced cellular antioxidant defense, as shown here by the lower levels of glutathione in response to ozone exposure. In addition, urban birds have relatively more ω6-to ω3-PUFAs circulating in their body (and presumably also a more ω6-rich diet) compared to rural birds (Andersson et al., 2015). Diet could therefore also influence the overall physiological state of urban birds, since experimental diet-type affected both the total antioxidant capacity and the response of the glutathione redox-system to ozone exposure. Yet, whether any of the two PUFA-diets would worsen or improve the overall condition of the urban birds remains to be unraveled.

## Conclusion

In summary, we found that diets differing in ω6-and ω3-PUFA concentrations modulate the overall plasma antioxidant capacity and redox state of the cellular glutathione system depending on ozone exposure. Ozone alone depleted the cells from glutathione, which in the long run may have severe consequences for the organism. However, only an immune challenge caused increased levels of oxidative damage, which is notable since it is an adaptive (yet apparently costly) part of the immune defense. Taken together, ozone exposure, dietary PUFAs, and infection simulation all influenced oxidative stress biomarkers of birds but through different physiological pathways. Disease, diet, and air pollution therefore appear as potential parallel drivers of the increased oxidative stress associated with urban-dwelling organisms. Additionally, our results indicate that the response to a simultaneous change of more than one anthropogenic pollutant may lead to different responses, compared to single stressor exposure. Since diet and disease prevalence co-occur with anthropogenic pollution in urban habitats, experimental studies using multiple stressors is key to understand the overall impact urbanization has on organismal health. Despite the lack of any apparent cumulative or synergistic effect of our stressors, the combined effect and conclusion from the independent biomarkers suggests that urban birds face an overall challenged physiological state.

## Data Availability

The raw data supporting the conclusion of this article will be made available by the authors, without undue reservation.
