# Supplementary material for: Dietary fatty acids modulate oxidative stress response to air pollution but not to infection
Source: Front Physiol. 2024 May 9;15:1391806. doi: 10.3389/fphys.2024.1391806 (PMC11112072; doi:10.3389/fphys.2024.1391806)
Supplement: Supplementary file 1 [file Presentation1.pdf]

Supplementary files
(Table S1-S5)

Dietary fatty acids modulate oxidative stress response to air pollution but not to infection

Ann-Kathrin Ziegler[*1], Johan Kjellberg Jensen [*1,2], Lucía Jiménez Gallardo[3], Jenny Rissler[4,5], Anders Gudmundsson[4], Jan-Åke Nilsson[1] & Caroline Isaksson[1^]

[1]Department of Biology, Lund University, 223 62 Lund, Sweden

[2]Centre for Environmental and Climate Science (CEC), Lund University, 223 62 Lund, Sweden

[3]Department of Biodiversity, Ecology and Evolution, Complutense University of Madrid, 28040 Madrid, Spain

[4]Ergonomics and Aerosol Technology, Department of Design Sciences, Lund University, 221 00 Lund, Sweden

[5]Bioeconomy and Health, RISE Research Institutes of Sweden, 223 60 Lund, Sweden

^Corresponding author: Caroline.Isaksson@biol.lu.se

*shared first-authorship

ORCID: A.-K.Z: 0000-0002-2593-0349; C.I.: 0000-0002-6889-1386; J.R.: 0000-0001-8650-474

Table S1. Number of zebra finches (*Taeniopygia guttata*) for which we have measurments of the different physiological assays and body mass. The sample sizes are shown for each of the four experimental weeks (two indepdent weeks with normal air and two independent weeks with ozone expsoure). x/y = number of birds that received ω6-rich diet/ number of birds that received ω3-rich diet. tGSH = total glutathione, GSH/GSSG = reduced/oxidized glutathione ratio, OXY = antioxidant capacity assay, dROM = oxidative damage assay.

	**tGSH (ω6/ω3)**	**GSH/GSSG (ω6/ω3)**	**OXY (ω6/ω3)**	**dROM (ω6/ω3)**	**Body mass**
Normal air	22/20	22/18	21/18	19/18	24/22
Ozone	24/22	23/22	21/23	20/21	24/23
Normal air	23/23	21/22	22/24	18/20	24/24
Ozone	22/23	22/22	23/20	23/16	24/23

Table S2. Table showing the experimental set-up and procedure. Day 0 the birds of each batch (week) were randomized into either ω6- or ω3-rich diet. At day 7 the air treatment started with either normal air (week 1 and 3) or ozone (week 2 and 4). In the morning of day 12, half of each diet treatment got either LPS or PBS injections. In the evening, 12 hours after the immune challenge a blood sample was taken.

**Normal air: week 1 and 3**	**Day -6**	**Day 0**	**Day 7**	**Day 12**	**Day 12**
**Birds brought indoors**					
**Diet treatment started** (half group got either ω6- or ω3-rich diet)					
**Birds brought to exposure chamber**					
**Start exposure** (normal air)					
**LPS or PBS injection** (half of each diet group got either LPS or PBS)					
**Blood sampling**					
**End exposure**					

**Ozone: week 2 and 4**	**Day -6**	**Day 0**	**Day 7**	**Day 12 (morning)**	**Day 12 (evening)**
**Birds brought indoors**					
**Diet treatment started** (half group got either ω6- or ω3-rich diet)					
**Birds brought to exposure chamber**					
**Start exposure** (ozone)					
**LPS or PBS injection** (half of each diet group got either LPS or PBS)					
**Blood sampling**					
**End exposure**					

Fatty acid analysis and calculation

We followed the protocol published in Andersson et al., (2018) to extract and analyse circulating fatty acids (FA), using 5 µl of plasma. In short, FA were extracted by using 50 µl chloroform:methanol (2:1 v/v). FA in the plasma were converted into FA methyl esters (FAMEs) by reaction with acidified methanol. FAMEs were measured by an Agilent 5975 mass spectrometer coupled to an Agilent 6890 gas chromatograph, equipped with an HP-INNOWax PEG column (30 m, 0.25 mm i.d., 0.25 mm film thickness; Agilent). FAMEs were identified by their retention times relative to standards and quantified using Agilent ChemStation software.

In total we identified 19 FAs. For each sample we calculated relative proportions of the individual FAs by dividing each peak area with the sum of all FA peaks. Furthermore, we calculated the ω-6/ ω-3 ratio of polyunsaturated FAs, as well as the level of FA unsaturation by calculating an index where the relative proportion of each individual FA was multiplied with their respective number of double bonds (Jezierska et al. 1982, Jensen et al. 2020).

Statistical analysis of the effects of differing dietary ω6:ω3 ratio of polyunsaturated FAs and exposure to elevated ozone levels was done using linear regression models with diet (high ω6:ω3 ratio or low ω6:ω3 ratio) and air treatment (normal air or ozone) as fixed effects and the respective FA proportion as the dependent variables. The proportions of eicosapentaenoic acid, arachidonic acid and alpha-linolenic acid were log-transformed to reach a normal distribution of the residuals. All statistical analysis were performed in R 4.1.0 (R CoreTeam, 2020).

Table S3. Summary of relative abundance of total circulating fatty acids (% of total fatty acid content) in female zebra finches (*Taeniopygia guttata*). Results are presented as the mean ± standard error of the mean. C:Dn-x = number of carbon atoms: number of double bonds - double bond position; SFA = saturated fatty acid; MUFA = monounsaturated fatty acid; PUFA = polyunsaturated fatty acid.

**Common name**	**C:D**	**FA group**	**ω-3, normal air (N=6)**	**ω-3, ozone (N=6)**	**ω-6, normal air (N=6)**	**ω-6, ozone (N=5)**	
Total SFA		SFA	56.1 ± 2.4	55.8 ± 3.2	50.8 ± 1.7	49.2 ± 2.2	**Diet: F=5.93, P=0.024**<br>Air: F=0.17, P=0.69
Total MUFA		MUFA	13.6 ± 2.0	14.6 ± 1.1	19.7 ± 2.8	14.7 ± 0.9	Diet: F=2.78, P=0.11<br>Air: F=0.85, P=0.37
Total PUFA		PUFA	30.3 ± 2.6	29.6 ± 2.8	29.5 ± 2.8	36.2 ± 0.18	Diet: F=0.92, P=0.35<br>Air: F=1.10, P=0.31
Total ω-3		ω-3 PUFA	3.8 ± 0.5	4.2 ± 0.5	0.4 ± 0.1	0.4 ± 0.1	**Diet: F=86.00, P<0.001**<br>Air: F=0.40, P=0.54
Total ω-6		ω-6 PUFA	26.5 ± 2.4	25.4 ± 2.4	29.1 ± 2.7	35.7 ± 1.8	**Diet: F=6.11, P=0.023**<br>Air: F=1.06, P=0.32
ω-6/ω-3 ratio		PUFA	7.47 ± 0.94	6.32 ± 0.60	101.83 ± 14.99	94.69 ± 13.28	**Diet: F=93.17, P<0.001**<br>Air: F=0.18, P=0.68
Unsaturation index			0.88 ± 0.06	0.90 ± 0.08	0.83 ± 0.05	0.93 ± 0.05	Diet: F=0.020, P=0.89<br>Air: F=0.83, P=0.37
Linoleic acid (LA)	18:2n-6	ω-6 PUFA	25.2 ± 2.2	24.2 ± 2.3	26.9 ± 2.4	32.7 ± 1.4	**Diet: F=4.72, P=0.042**<br>Air: F=1.00, P=0.33
Alpha-linolenic acid (ALA)	18:3n-3	ω-3 PUFA	0.2 ± 0.1	0.2 ± 0.0	0.1 ± 0.0	0.1 ± 0.0	(log-transf.)<br>**Diet: F=5.08, P=0.034**<br>Air: F=0.25, P=0.62
Arachidonic acid (AA)	20:4n-6	ω-6 PUFA	0.8 ± 0.1	0.8 ± 0.1	1.7 + 0.3	2.4 ± 0.5	(log-transf.)<br>Diet: F=28.14, **P < 0.001**<br>Air: F=1.38, P=0.25
Docosahexaenoic acid (DHA)	22:6n-3	ω-3 PUFA	1.1 ± 0.1	1.3 ± 0.2	0.1 ± 0.0	0.2 ± 0.0	**Diet: F=73.12, P<0.001**<br>Air: F=1.23, P=0.28
Eicosapentaenoic acid (EPA)	20:5n-3	ω-3 PUFA	2.4 ± 0.3	2.6 ± 0.3	0.05 ± 0.0	0.1 ± 0.0	(log-transf.)<br>**Diet: F=115.13, P<0.001**<br>Air: F=0.22, P=0.65

Table S4. Dropped factors from final linear mixed models, testing responses of total gluathione (tGSH), ratio of reduced/oxidized gluathione (GSH/GSSG), non-enzymatic antioxidant capacity (OXY), and oxidative damage levels (ROMs) of zebra finches (*Taeniopygia guttata*) after a five day experimental exposure to either ozone or normal air (air treatment), while feeding on either of the two differential fatty acid diets (ω6-rich or ω3-rich) and subsequent immune challenge (phosphate-buffered saline [PBS] injection or lipopolysaccharide [LPS] injection).

**Post-exposure tGSH concentration (µM)**		
**Dropped terms**	**Chisq**	**P**
Diet x immune challenge x air treatment	0.116	0.73
Immune challenge x air treatment	$8e^{-4}$	0.98
Immune challenge x diet	0.258	0.61
Replicate	0.477	0.49
Sex	0.679	0.41
Diet x air treatment	1.635	0.20
**Post-exposure GSH/GSSG ratio**		
**Dropped terms**	**Chisq**	**P**
Centred body mass	0.04	0.84
Sex	0.770	0.38
Diet x immune challenge x air treatment	1.556	0.21
Immune challenge x air treatment	1.523	0.22
Immune challenge x diet	2.147	0.14
Replicate	2.457	0.12
**Post-exposure antioxidant capacity (OXY)**		
**Dropped terms**	**Chisq**	**P**
Replicate	0.002	0.97
Diet x immune challenge x air treatment	0.176	0.68
Immune challenge x air treatment	0.046	0.83
Immune challenge x diet	0.339	0.56
Diet x air treatment	0.677	0.41
Sex	0.863	0.35
**Post-exposure oxidative damage (log-transformed dROMs)**		
**Dropped terms**	**Chisq**	**P**
Diet x immune challenge x air treatment	0.890	0.34
Immune challenge x air treatment	0.844	0.36
Replicate	1.417	0.23
Diet x air treatment	1.531	0.22
Immune challenge x diet	1.735	0.19

Table S5. Final models and dropped factors from linear mixed models, testing responses of post-exposure body mass of zebra finches (*Taeniopygia guttata*) after a five-day experimental exposure to either ozone or normal air (air treatment), while feeding on either of the two differential fatty acid diets (ω6-rich or ω3-rich) and subsequent immune challenge (phosphate-buffered saline [PBS] injection or lipopolysaccharide [LPS] injection).

**Body mass (post-exposure)**					
**Random effects**	**Variance**	**StDev**	**# groups**	**# observations**	
Experimental cage	0.068	0.260	8	179	
Residual	3.162	1.778			
**Fixed effects**	**Estimate ± SE**	**SS**	**df**	**F**	**P**
Intercept	14.107 ± 0.302				
**Replicate (2)**	**0.768 ± 0.259**	**27.715**	**1, 176.34**	**8.764**	**0.003**
Diet (ω3)	-0.131 ± 0.259	0.802	1, 176.26	0.254	0.615
Immune challenge (LPS)	-0.030 ± 0.260	0.042	1, 176.38	0.013	0.908
Air treatment (ozone)	0.124 ± 0.260	0.718	1, 176.38	0.227	0.634

**Dropped terms**		
**Fixed effects**	**Chisq**	**P**
Diet x immune challenge x air treatment	0.225	0.635
Sex	1.066	0.302
Diet x air treatment	1.643	0.200
Immune challenge x diet	2.480	0.115
Immune challenge x air treatment	3.013	0.083

References

Andersson, M. N., J. Nilsson, J.-Å. Nilsson, and C. Isaksson. 2018. Diet and ambient temperature interact to shape plasma fatty acid composition, basal metabolic rate and oxidative stress in great tits. The Journal of Experimental Biology 221:jeb186759.

Jensen, J. K., C. Isaksson, C. Eikenaar, and M. N. Andersson. 2020. Migrant blackbirds, Turdus merula, have higher plasma levels of polyunsaturated fatty acids compared to residents, but not enhanced fatty acid unsaturation index. Ecology and Evolution 10:10196–10206

Jezierska, B., J. R. Hazel, and S. D. Gerking. 1982. Lipid mobilization during starvation in the rainbow trout, Salmo gairdneri Richardson, with attention to fatty acids. Journal of Fish Biology 21:681–692.

R CoreTeam, 2020. R: A language and environment for statistical computation. R Foundation for Statistical Computing, Vienna. https://doi.org/10.18637/jss.v067.i01
